# Poly(lactic acid) Composites Containing Carbon-Based Nanomaterials: A Review

**DOI:** 10.3390/polym9070269

**Published:** 2017-07-06

**Authors:** Carolina Gonçalves, Inês C. Gonçalves, Fernão D. Magalhães, Artur M. Pinto

**Affiliations:** 1 LEPABE—Faculdade de Engenharia, Universidade do Porto, rua Dr. Roberto Frias, 4200-465 Porto, Portugal; carol.goncalves8827@gmail.com (C.G.); fdmagalh@fe.up.pt (F.D.M.); 2 INEB—National Institute of Biomedical Engineering, University of Porto, Rua do Campo Alegre, 823, 4150-180 Porto, Portugal; icastro@ineb.up.pt; 3 i3S—Institute for Innovation and Health Research, University of Porto, Rua Alfredo Allen, 208, 4200-135 Porto, Portugal

**Keywords:** PLA, graphene-based materials, carbon nanotubes, composites, mechanical properties, thermal properties, electrical properties, biological properties

## Abstract

Poly(lactic acid) (PLA) is a green alternative to petrochemical commodity plastics, used in packaging, agricultural products, disposable materials, textiles, and automotive composites. It is also approved by regulatory authorities for several biomedical applications. However, for some uses it is required that some of its properties be improved, namely in terms of thermo-mechanical and electrical performance. The incorporation of nanofillers is a common approach to attain this goal. The outstanding properties of carbon-based nanomaterials (CBN) have caused a surge in research works dealing with PLA/CBN composites. The available information is compiled and reviewed, focusing on PLA/CNT (carbon nanotubes) and PLA/GBM (graphene-based materials) composites. The production methods, and the effects of CBN loading on PLA properties, namely mechanical, thermal, electrical, and biological, are discussed.

## 1. Introduction

The growing environmental awareness and new rules and regulations are forcing the industries to seek more ecologically friendly materials for their products [1]. In the last two decades, industrial and academic research on polymer composites was pursued to provide added value properties to the neat polymer without sacrificing its processability or adding excessive weight [2]. 

Poly(lactic acid) (PLA), which is derived from natural sources, biodegradable, and bioabsorbable, has had significant demand due to presenting versatile applications in packaging, pharmaceutical, textiles, engineering, chemical industries, automotive composites, biomedical and tissue engineering fields [3]. Its biodegradation time can be tuned, depending on the molecular weight, crystallinity, and material geometry [4]. However, the relatively low glass transition temperature, low thermal dimensional stability, and mechanical ductility limit the number of its applications. A significant body of research has dealt with the use of fillers for improving the properties of PLA [5,6,7]. In this context, carbon based nanomaterials (CBN), offer the potential to combine PLA properties with several of their unique features, such as high mechanical strength, electrical conductivity, thermal stability and bioactivity [8,9,10,11,12,13,14,15,16]. Carbon nanotubes (CNT) and graphene-based materials (GBM) are state of the art and very promising representatives of these materials. CNT have exceptional mechanical properties, aspect ratio, electrical and thermal conductivities, and chemical stability. However, their production methods are usually more complex and expensive, often leaving toxic metal residues [17,18,19,20]. Hence, GBM provide an alternative option to produce functional composites due to their excellent properties and the natural abundance of their precursor, graphite. Moreover, GBM can be produced by simple and inexpensive physico-chemical methods [21,22,23,24].

In the last years there has been a surge of research works on PLA/CNT and PLA/GBM composites. Due to the large amount of information available, there is the need to congregate, compare and withdraw conclusions.

Several recent reviews have addressed PLA [3,25,26,27,28,29,30] and CBN [30,31,32,33,34,35,36,37,38,39,40,41,42,43,44,45,46] production, applications and properties, however, none of these focus on PLA/CBN composites. This work presents a comprehensive review on the current knowledge regarding the production of PLA/CBN composites and the resulting properties, namely mechanical, electrical, thermal and biological.

## 2. Poly(lactic acid) (PLA)

PLA is a thermoplastic aliphatic polyester commonly produced by direct condensation polymerization of lactic acid or by ring-opening polymerization of lactide. As lactic acid is a chiral molecule, existing in l and d isomers, the term “poly(lactic acid)” refers to a family of polymers: poly-l-lactic acid (PLLA), poly-d-lactic acid (PDLA), and poly-d,l-lactic acid (PDLLA). The 2 optically active configurations of lactic acid, the l (+) and d (−) stereoisomers are produced, respectively by bacterial homo- or hetero-fermentation of carbohydrates. A great variety of carbohydrate sources can be used to produce lactic acid, like molasses, corn syrup, whey, dextrose, and cane or beet sugar. Nowadays, industry only uses the fermentation process, because the synthetic routes have major limitations, as the inability of selective production of the l-lactic acid stereoisomer, and high manufacturing costs [47,48].

PLA can be polymerized by diverse methods, like polycondensation, ring opening polymerization, azeotropic dehydration condensation, and enzymatic polymerization. Direct polymerization and ring opening polymerization are the most used. Controlling polymerization parameters is important, since PLA properties vary with isomer composition, temperature, and reaction time used [3,25,28,29,48,49,50,51].

Increasing interest in PLA is related to some characteristics that are lacking in other polymers, namely regarding renewability, biocompatibility, processability, and energy saving [29]. PLA is derived from renewable and biodegradable resources, and its degradation products are non-pollutant and non-toxic. Thus, PLA is a green alternative to petrochemical commodity plastics, used in packaging, agricultural products, disposable materials, textiles, and automotive [25]. Furthermore, PLA has several bioapplications, such as biodegradable matrix for surgical implants, and in drug delivery systems [3].

The use of PLA has some shortcomings, related to poor chemical modifiability (absence of readily reactive side-chain groups), mechanical ductility [50], and relatively high price [28]. To overcome some of these issues, some approaches are commonly used, like blending with other polymers [52,53,54,55,56,57,58,59], functionalization [60,61,62,63,64], and addition of nanofillers [6,7,48,65,66,67,68,69,70]. The last is an interesting approach, since with small filler amounts it is possible to enhance desired features, keeping PLA’s key properties intact. The most used nanofillers are nanoclays [5,71,72,73,74,75,76,77,78,79,80], nanosilicas [6,68,69,73,81,82], and carbon nanomaterials [7,77,83,84,85,86,87,88].

## 3. Carbon-Based Nanomaterials (CBN)

There are several types of carbon-based nanomaterials (carbon nanotubes, graphene-based materials, fullerenes, nanodiamonds) and most have been tested to improve PLA properties. This review is focused on the most widely tested and available: CNT and GBM. The high specific area of these materials allows for low loadings to be sufficient to tune key properties concerning mechanical, thermal, electrical, and biological performance.

### CBN Production Methods and Modifications

Graphene is the elementary structure of graphite, being a one carbon atom thick sheet, composed of sp^2^ carbon atoms arranged in a flat honeycomb structure composed of two equivalent sub-lattices of carbon atoms bonded together with σ bonds (in plane) and a π bond (out-of-plane), which contributes to a delocalized network of electrons [39,46,89]. These unique characteristics explain its unmatched electronic, mechanical, optical and thermal properties. For that reason, this material has been studied to be applied in many fields, such as electronics [90,91,92,93,94,95], energy [96,97,98,99], membrane [100,101,102,103], composite [21,22,24,104], and biomedical technology [11,105,106,107].

The intrinsic properties of graphene, and GBM in general, are affected by the production or modification methods. For example, structural integrity of graphene sheets is disrupted by oxidation and some other chemical modifications. The dimensions (diameter and thickness) of the final GBM also depend on the raw materials and methods employed [11,34,35,46,90]. Thus, those should be chosen according to desired applications.

GBM can be obtained by top-down and bottom-up approaches [104]. The first involves exfoliating graphite to obtain few or single layer graphene sheets [38,108]. The second, consists in assembling graphene from deposition of carbon atoms from other sources [109,110]. The main difficulty in top-down methods is to overcome the van der Waals forces that hold the graphene layers together in graphite, preventing reagglomeration and avoiding damages in the honeycomb carbon structure [111,112]. Some examples of such methods are micromechanical exfoliation, direct sonication, electrochemical exfoliation, and superacid dissolution. Bottom-up methods include chemical vapor deposition (CVD), arc discharge, and epitaxial growth on silicon carbide [104]. 

The structure of CNT can be conceptualized by wrapping graphene into a cylinder. Typically, CNT are classified as either single-walled carbon nanotubes (SWCNT) or multi-walled carbon nanotubes (MWCNT). SWCNT exhibit better electrical properties, while MWCNT display better chemical resistance [113].

CNT can be produced using different methods, which mainly involve gas phase processes [114,115], like CVD, arc discharge, and laser ablation [116]. The most commonly used and efficient methods are the ones involving CVD, in which a carbon containing source (e.g., methane, acetylene, ethylene) reacts with a metal catalyst particle (e.g., iron, cobalt, nickel) which act as growth nuclei for CNT, at temperatures above 600 °C. There are several substrate materials for catalyst particles, as graphite, quartz, silicon, silicon carbide, amongst others. It is pertinent to mention that for graphene production by this technique, no catalyst particles are used, being the substrate itself a catalytic metal, often copper for monolayer or nickel for few layer graphene. Generally, CVD has the advantages of allowing mild and controllable synthesis in large scale [117,118,119,120].

CNT are strong, flexible, electrically conductive, and can be functionalized [121]. Potential applications of CNT have been reported such as in composite materials [122], electrochemical devices [123], hydrogen storage [124], field emission devices [125], nanometer-sized electronic devices, sensors and probes [126]. Determining the toxicity of CNT has been one of the most pressing questions in nanotechnology [127]. There is still some controversy on this subject, thus continued research is needed to assure that these materials are safe for biomedical applications [128,129]. Parameters such as structure, size distribution, surface area, surface chemistry, surface charge and agglomeration state, as well as the sample purity, have considerable impact on CNT properties [121].

**Figure 1:**
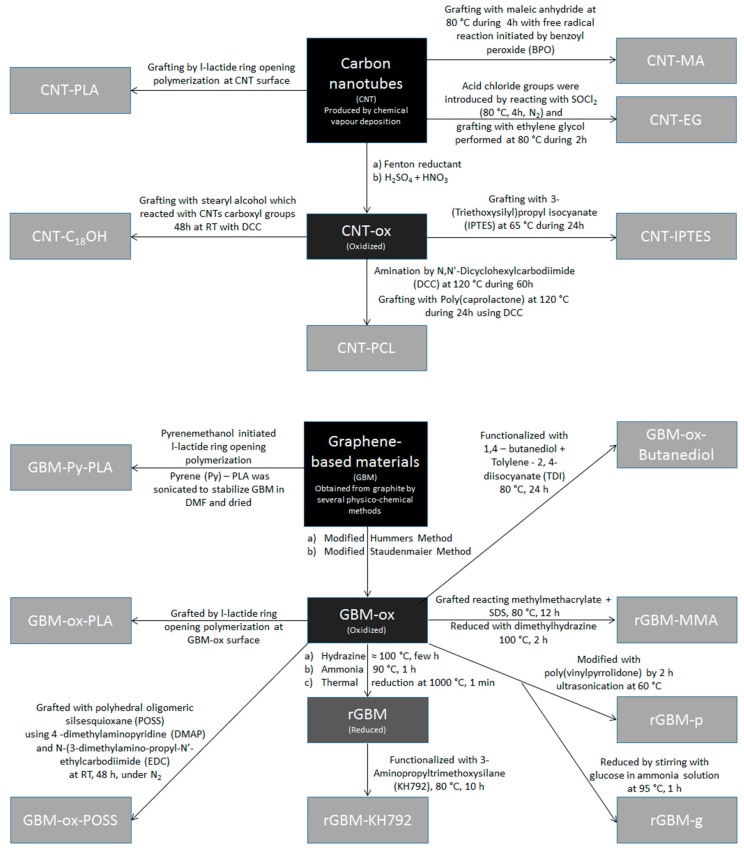
Scheme showing the different types of modifications performed on carbon-based nanomaterials (CBN) prior to incorporation in poly(lactic acid) (PLA).

In the research works reported in this review, CBN are both commercial products or lab-made by the authors. Most commercial CNT are produced by CVD, with suppliers often making available information about material dimensions and sometimes type of CVD used. On the other hand, researchers usually produce GBM from graphitic precursors, using top-down methods involving chemical oxidation and exfoliation, namely the Staudenmaier and modified Hummers methods (Figure 1). Commercial GBM are also used, with suppliers giving information about dimensions, and sometimes production methods. These involve direct exfoliation in a liquid, with or without the use of a surfactant, or in the solid state by edge functionalization, or by first inserting a chemical species between the graphene layers in graphite to weaken their interaction, followed by expansion/exfoliation [130]. Commercial products offer insured reproducibility and widespread availability. Moreover, with the optimization of the production processes, the costs of GBM are coming closer to its precursor, graphite [11].

CBN have been extensively used in polymer composites. In order to take advantage of their large surface area maximizing its effectiveness as filler, dispersion must be efficient, so as to maximize the amount of deagglomerated primary units. Functionalization is often used to improve compatibility with the polymer matrix. However, this can disrupt the sp^2^ hybridization of CBN carbon structure and subsequently hinder their properties [131]. Some examples of CBN modifications used on the research works reported in this review are compiled in Figure 1. Some of these involve simple chemical oxidation, prior to surface modification with isocyanates, polymers (ethylene glycol, poly(caprolactone), methyl methacrylate, poly(vinyl pyrrolidone), and PLA), polyols or silanes. The impact of these on the composite properties is discussed in Section 5.

## 4. Production of PLA/CBN Composites

Three methods are most frequently used to obtain a dispersion of CBN into a polymer matrix: solution mixing, melt blending, and in situ polymerization [22,104]. Mechanical milling, also called ball milling, has been gaining recognition as an alternative technique with specific advantages, but it has not yet been reported for PLA/CBM composites. High impact milling is performed at room temperature on dry powders, prior to melt processing. Its effectiveness and benefits in relation to other methods have been shown for different polymer/filler systems [132].

### 4.1. Solution Mixing

Solution mixing is a simple procedure, requiring no special equipment, and allowing for straightforward scale-up. This method typically consists of three steps: (i) dispersion of the nanomaterial in a suitable solvent using sonication or mechanical stirring; (ii) dissolution of the polymer in the previous dispersion, under appropriate stirring; and (iii) removal of the solvent by distillation or lyophilization. Often the dispersion is cast into a flat mold, and then the solvent is evaporated. Flat composite slabs are therefore obtained. For this reason, the procedure is often called “solvent casting”. As an alternative, the dispersion may be cast onto a low surface energy material (e.g., PTFE coated surface) using a blade applicator (doctor blading). After solvent evaporation, thin composite films are obtained. The viscosity of the dispersion needs to be adjusted for this procedure, which can be done by changing the concentration of polymer [133]. If production of fibers is desired, the third step can be replaced by electrospinning. This technique allows obtaining fibers that are much smaller in diameter (ranging from micrometers to nanometers) than those produced by conventional techniques. The basis of electrospinning is to charge the polymer solution in the spinneret tip with a high voltage, so that the electrostatic repulsion overcomes the surface tension of the solution, causing its ejection. The solvent vaporizes while the jet is in the air, producing a continuous fiber which deposits on the ground collector [27].

Complete solvent removal is a critical issue when using solution mixing to prepare composites, since toxicity concerns may arise when organic solvents are used. In addition, presence of residual solvent induces plasticization of the polymer matrix, which may alter significantly its mechanical properties [134,135,136].

PLA is soluble in organic solvents such as chlorinated solvents, benzene, tetrahydrofuran (THF), dimethyl formamide (DMF) and dioxane, but insoluble in ethanol, methanol, and aliphatic hydrocarbons. CBN are hydrophobic, therefore cannot be easily dispersed in polar solvents. However, they can be oxidized or modified with hydrophilic groups in order to allow dispersion in such solvents. Solubility limitations can also be overcome to a certain point by using ultrasonication to produce short-time metastable dispersions of CBN in organic solvents, which can then be mixed with polymer solutions [137].

Chloroform is the most used solvent to prepare PLA/CNT composites [138,139,140,141,142,143]. Despite, some authors obtain good results with THF [88,144], and dichloromethane [145,146]. McCullen et al. [147] conclude that a combination of chloroform and DMF is beneficial. Sometimes the introduction of new functional groups may originate incompatibility with the polymer matrix. To elude this problem, improvement of CNT dispersion by surfactant addition (e.g., polyoxyethylene 8 lauryl, dodecyl octaethylene) may be used, which allows preserving the chemical structure of the nanofiller [148]. GBM have been often incorporated in PLA by solution mixing using chloroform [135,149,150,151] or DMF [152,153,154,155,156,157] as solvents. Agglomeration of CBN may take place during solvent evaporation. Composite formation by electrospinning allows minimizing this problem, but leads to formation of fibers and not films [27,147,158].

### 4.2. Melt Blending

Melt blending is an economically attractive, environmentally friendly and highly scalable method for preparing nanocomposites. This strategy involves direct addition of the nanomaterial into the molten polymer, allowing optimization of the state of dispersion by adjusting operating parameters such as mixing speed, time and temperature. Due to the absence of solvent, the only compatibility issue is placed in terms of the nanofiller towards the polymer matrix [27,48]. The drawbacks of this procedure are the low bulk density of CBN, that makes the feeding of the melt-mixer a troublesome task and the lower degree of dispersion that is usually attained when compared to solvent mixing [137,159].

Most published research works use a lab-scale melt mixer to melt PLA and mix it with the nanofillers. Typical processing conditions correspond to temperatures between 160 °C and 180 °C [160,161,162,163,164,165,166], mixing times of 5 to 10 min [160,161,162,164,165,167], and rotation speeds between 50 and 100 rpm [160,161,162,163,164,166,167,168,169]. After mixing, the composite materials are almost always molded into flat sheets with controlled thickness in a hot press, however, other methods are also used (e.g., injection molding and piston spinning). Typically, the pressing is performed between 160 °C and 190 °C for 2 to 5 min, under 110 to 150 Kgfcm^−2^ pressure [160,165,166,167,168,169,170].

In addition to melt blending not being as effective as the solution mixing method or in situ polymerization in terms of the ability to achieve good filler dispersion, damage to the nanofillers or polymer may occur under severe conditions. Some studies have shown that processing conditions can have an impact on the molecular weight of PLA [171]. This can be mainly attributed to the presence of impurities such as acidic species, peroxide groups, metallic ions or other residual products that can increase the degradation of PLA during melt mixing [172].

### 4.3. In Situ Polymerization

In situ polymerization for production of polymer composites generally involves mixing the filler in neat monomer, or a solution of monomer, in the presence of catalysts and under proper reaction conditions [173]. The polymer chains grow on the filler surface, being covalently bonded. In situ polymerization generally results in more homogeneous particle dispersion than melt blending [174]. Use of this approach for polymerizing lactide in the presence of CNT has been reviewed by Brzeziński and Biela [175]. Contrary to CNT, that usually are post-treated, GBM already present some chemical groups that can be used in further functionalization, such as grafting polymer chains via atom transfer radical polymerization. Examples of in situ polymerization on GBM include polymers such as polyaniline (PANI), polyurethane (PU), polystyrene (PS), poly(methyl methacrylate) (PMMA) and polydimethylsiloxane (PDMS) [24].

Concerning the particular case of PLA/CBN, only a few examples of in situ polymerization can be found in the literature. Ring opening polymerization of l-lactide in presence of GBM has been reported by Yang et al. [176] and Promoda et al. [177]. Carboxyl-functionalized CNT have been grafted with PLA by Li and co-workers [178].

**Figure 2:**
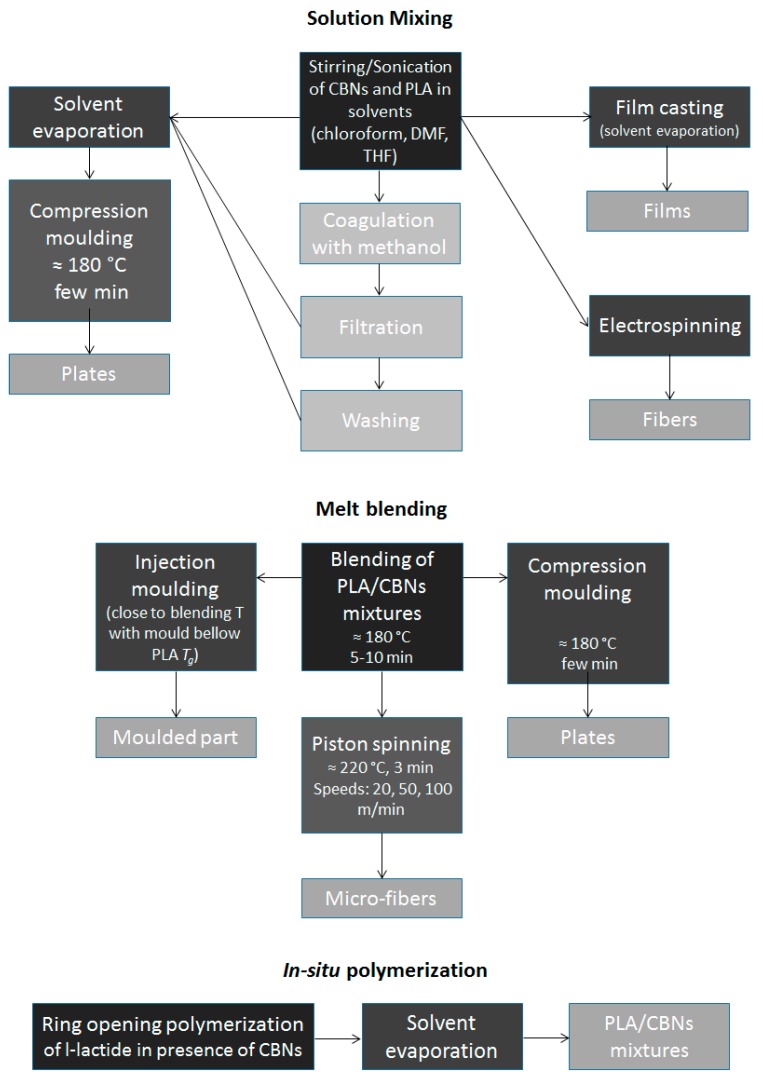
Scheme showing the different production methods of PLA/CBN composites.

The above-mentioned composite production methods can be used both with GBM and CNT, and are congregated in Figure 2. 

## 5. Properties of PLA/CBN Composites

Numerous researchers have studied the properties of PLA combined with other materials, in order to tune key properties regarding specific applications [48]. The current review is focused on the effect of incorporating two carbon-based nanomaterials, CNT and GBM, in PLA. CNT are known for two decades and have well established large-scale production methods. GBM, which have been raising a growing interest from the scientific community, are cheaper and, in principle, comparable in properties to CNT [177].

### 5.1. Mechanical Properties

**Figure 3:**
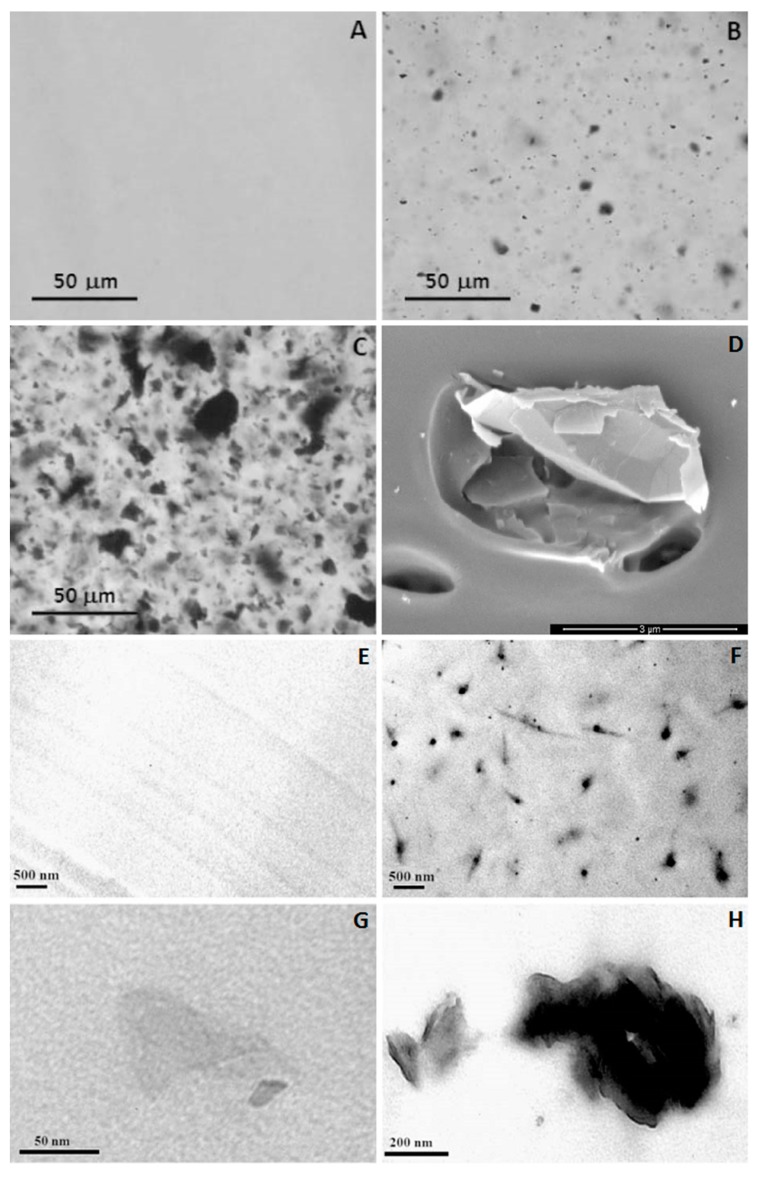
Microscopy images of PLA, PLA/GNP and GO 0.4 wt % films produced by solution mixing followed by film casting using doctor blading, displaying good filler dispersion and interaction with polymer matrix. Optical microscopy images of PLA (**A**); PLA/GO (**B**); and PLA/GNP (**C**); Scanning electron microscopy image of PLA/GNP (**D**); Transmission electron microscopy images of PLA (**E**) and PLA/GO (**F**–**H**) [179].

Physico-chemical interactions between fillers and polymer phase contribute to load transfer and distribution along the CBN network. Table 1 shows that solution mixing is the most commonly reported method for incorporation of CBN in PLA. The most frequently used solvents are chloroform, DMF and THF. The filler concentrations most often tested are between 0.1–2 wt %. Maximum improvements in Young’s modulus (*E*), storage modulus (*E’*), and tensile strength (*σ_max_*) are found for concentrations between 0.25–5 wt % for CNT, and between 0.1–1 wt % for GBM. The larger improvement in *E*, relative to unfilled PLA, is of 372%, for 0.25 wt % MWCNT sonicated in a PLA/chloroform dispersion, followed by compression molding of the dried mixture [138]. For GBM, the best performance is an increase of 156% with incorporation of 0.4 wt % GNP-M, also by sonication, but followed by film casting using doctor blading. In this study, comparison is made with GO, which yields a maximum *E* increase at 0.3 wt % loading. Figure 3 presents microscopy images demonstrating good dispersion of the fillers in the PLA matrix [135]. 

The maximum increase on *E’* is of 1500%, achieved with incorporation of 0.5 wt % rGO-KH792 in PLLA, by simple stirring, casting on PTFE mold, and vacuum drying the resultant films at 120 °C for 48 h [157]. However, this increase only occurs around PLA transition temperature (60–65 °C). At ambient temperature, the best result is an increase of 67% with incorporation of 3 wt % A-SWCNT-Si (acid treated and grafted with 3-isocyanatoporpyl triethoxysilane) in PLA by sonication, followed by drying and compression molding at 190 °C [144]. The maximum increase in *σ_max_* is of 129 wt %, obtained with incorporation of 0.4 wt % GNP-M in PLA by sonication and film casting by doctor blading [135]. For CNT the best result is an increase of 47% obtained with MWCNT grafted with PLA, and then incorporated at a loading of 1 wt % in PLA by sonication in chloroform, separation, drying and compression molding at 180 °C [141]. When considering CNT without modification, the best result reported is an increase of 9% for 1.2 wt % MWCNT incorporated in PLA by solution mixing, followed by drying and compression molding at 180 °C with a pressure of 1000 Kg [142].

Melt-blending is less frequently reported than solution mixing for production of PLA/CBN composites, probably due to the lower availability of the necessary equipment. Results show that it tends to be not as effective in improvement of mechanical properties, as solution mixing. The best performance in terms of *E* (↑88%) and *E’* (↑76%) is reported by Lin et al. [160] for an incorporation of 3 wt % MWCNT grafted with stearyl alcohol (MWCNT-C_18_OH) in PLA by melt blending (180 °C, 5 min, 50 rpm), using Ti(OBu)_4_ for transesterification, followed by compression molding at the same temperature. When PLA is not transesterified, *E* and *E’* increases were of 74% and 44%, respectively. The maximum increase in *σ_max_* (40%) is obtained incorporating 0.08 wt % rGO using a twin-screw mixer (175 °C, 8 min, 60 rpm), followed by compression molding at 180 °C [168]. The incorporation by melt blending (180 °C, 20 min, 50 rpm) of 0.25 wt % GNP-M5 and C in PLA followed by compression molding at 190 °C, prevented its mechanical properties decay after 6 months degradation in phosphate-buffered saline at 37 °C [180].

In situ polymerization is the least used technique. It has been reported by Pramoda et al. [177], who performed PLA ring-opening polymerization in presence of 1 wt % of GO functionalized with butanediol and GO modified with POSS silsesquioxane. In the first case, improvements of 1% and 14% in *E* and hardness are obtained, respectively. In the second, the performance is increased by 33% and 45%, in the same order.

Comparing the results for CNT and GBM, we can conclude that both can effectively improve PLA mechanical properties, whether by solution mixing and melt blending. However, use of GBM usually implies lower amounts of GBM than of CNT. Several chemical modifications have been tried to improve compatibility with the polymer matrix, with ineffective results is some cases. Functionalization with carboxyl groups is the most common and effective procedure to improve CNT compatibility with PLA matrix [146]. On the other hand, no relation has been observed between CBN morphological properties (size, length, and diameter) and the mechanical performance of the composites.

### 5.2. Electrical Properties

Neat PLA is electrically insulating with a low electrical conductivity (*σ* ≈ 1 × 10^−16^ S m^−1^), and high sheet resistance (*ρ_□_* ≈ 5 × 10^12^ Ω sq^−1^) [144,160]. Since CNT and reduced forms of GBM present high electrical conductivity, they can be incorporated in PLA to improve its conductivity. This sort of composites have potential to be used as electrical stimulating implants, since PLA is used as a biodegradable matrix in orthopedic material. Other advantages of increasing PLA conductivity are the possibility of using it as antistatic coating/material or for electromagnetic shielding [104]. The minimum amount of filler required to form a conductive network within the polymer is called percolation threshold, and should be as low as possible in order to keep processing simple (relatively low viscosity of the melt) and low costs. Table 2 shows that, once again, the most used method to incorporate CBN on PLA for electrical properties evaluation is solution mixing. The amount of fillers ranges from 0.01 to 10 wt %. The best result, considering electrical conductivity (*σ*) with CNT is 3.5 × 10^−3^ S m^−1^, obtained incorporating 10 wt % MWCNT in PLA by sonication in chloroform, followed by drying and compression molding at 200 °C during 15 min [138]. Results are also often presented in terms of sheet resistance (*ρ_□_*), being the lowest value reported by Shao et al. [183], of 1 × 10^2^ Ω sq^−1^ achieved incorporating 5 wt % MWCNT previously oxidized (treated with HCl and HNO_3_) in PLA by solution mixing, followed by electrospinning of aligned nanofibers (d ≈ 250 nm). The alignment of the fibers slightly improved sheet resistance, comparing with random meshes. Interestingly, Yoon et al. [143] observe a considerable sheet resistance of 1 × 10^5^ Ω sq^−1^, with incorporation of 1 wt % MWCNT-COOH, also oxidized by treatment with strong acids (H_2_SO_4_ and HNO_3_). For GBM, the maximum conductivity reported is 2.2 S m^−1^, higher than for CNT, obtained incorporating 1.25 wt % rGO-g (reduced with ammonia) in PLA by sonication in DMF. Interestingly, the solvent used for dispersion of CNT in PLA is always chloroform and for GBM is always DMF.

**Figure 4:**
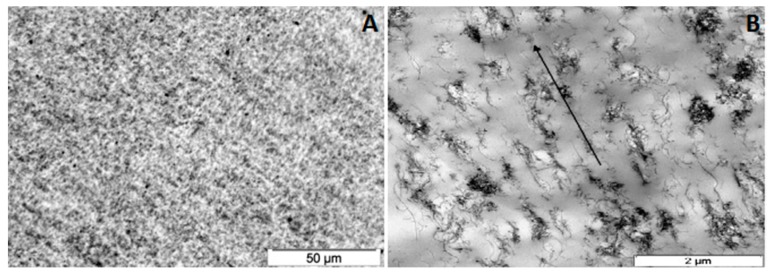
Optical microscopy image of a PLA/MWCNT 3 wt % mixture produced by twin-screw extrusion (**A**)—illustrating the high degree of macroscopic filler dispersion. Transmission electron microscopy image of a PLA/MWCNT 3 wt % mixture produced by twin-screw extrusion, followed by piston spinning; (**B**)—arrow indicates that fillers are strongly oriented in fiber direction due to the spinning process [185].

Melt-blending is the second most used approach to disperse CBN in PLA in order to improve its electrical properties, being most often performed by twin-screw extrusion, followed by compression molding. The highest *σ* considering CNT is 50 S m^−1^, which is reported by Pötschke et al. [184]. These authors prepare MWCNT mixtures by twin-screw extrusion, followed by piston spinning at different speeds. They conclude that non-spun mixtures with 5 wt % MWCNT in PLA present the same conductivity as 3 wt % mixtures after piston spinning at a speed of 20 m min^−1^. Microscopy images in Figure 4 allow to observe good MWCNT dispersion and orientation due to spinning process.

Considering *ρ_□_*, the best performance is obtained incorporating 3 wt % MWCNT-C_18_OH (MWCNT modified with DCC and stearyl alcohol) using and external mixer, followed by compression molding at 180 °C during 5 min, resulting in a *ρ_□_* of 1 × 10^−1^ Ω sq^−1^ [160]. This is the most effective modification performed, considering the sheet resistance values obtained with incorporation of the same amount of non-modified MWCNT, which was 3 × 10^5^ Ω sq^−1^. For GBM, the higher *σ* is 2.6 × 10^−4^ S m^−1^, resultant from dispersion using an internal mixer at 180 °C, of 5 wt % PFG (graphene nanoparticles functionalized with methylmethacrylate) [164]. For rGO, a non-functionalized GBM, the best conductivity value is obtained for 2 wt % incorporation in PLA using a twin-screw extruder and compression molding. The value obtained is of 1 × 10^−9^ S m^−1^, being higher than for the other concentrations tested. It can be compared, for example, with a *σ* of 1 × 10^−13^ S m^−1^ for 0.2 wt % [168]. In most works evaluated, electrical properties improve with the increase of filler amount.

In situ polymerization is the least explored technique, despite interesting results being obtained by Yang et al. [176], which incorporate 0.01–2 wt % trGO (thermally reduced) in PLA by ring-opening melt polymerization of l-lactide in presence of the filler. As example, *σ* obtained is 5 × 10^−6^ and 1.6 × 10^−2^ S m^−1^ for 1.5 and 2 wt %, respectively.

An interesting study by Chiu et al. [88], shows that purification of MWCNT by sonication with strong acids improved fillers compatibility and dispersibility in PLA, resulting in better electrical conductivity. The values of *σ* for incorporations of 7 wt % are 5 × 10^−8^ and 2 × 10^−6^ S m^−1^, respectively for non-purified and purified MWCNT. Purification introduced polar functional groups on the CNT surface, allowing better dispersion, which resulted in more deagglomerated particles that formed a wider conductive network on PLA matrix.

### 5.3. Thermal Properties

Several works studied thermal properties of PLA containing CBN. CNT incorporations range from 0.01 to 15 wt %, while for GBM lower amounts are needed 0.01–2 wt % (Table 3). However, for both CBN, slight or no changes are observed in the composites’ thermal properties, especially when low fillers amounts are used [135,146,156,157,160,161,162,167]. The most frequently used techniques to evaluate thermal properties in polymer composites are thermogravimetric analysis (TGA), differential scanning calorimetry (DSC), and dynamic mechanical analysis (DMA). TGA allows determination of thermal degradation temperatures (*T_d_*) and DSC and DMA phase transition temperatures (*T_g_*—glass transition temperature, *T_m_*—melting temperature, and *T_c_*—cold crystallization temperature).

A positive deviation in *T_d_* is expected when there is good compatibility between CBN and the polymer matrix, combined with good dispersion of the fillers. This leads to restriction of PLA’s chains motions, delaying thermal decomposition. Also, CBN can induce the formation of a crystallization region on their surfaces, which absorbs some heat as temperature of the composite increases. However, the incorporation of too high amounts of CBN can lead to the formation of agglomerates, which represent structural defects in the matrix, decreasing thermal stability [145]. Some works also attribute improvements in thermal stability to the barrier effect caused by the CBN, which creates a “tortuous path” delaying permeation of oxygen and the escape of volatile degradation products, and also to char formation [146,150,167]. Increases in *T_g_* are usually also associated with good interaction between CBN and polymer matrix, leading to constraint of PLA’s molecular mobility by hydrogen bonding and electrostatic attraction [139,140,146,150]. *T_m_* increases are usually attributed to a nucleation effect caused by the CBN, which increases the degree of crystallinity [146,150,176]. For the same reason, *T_c_* usually decreases with CBN incorporation [141,146,153,162,170,176]. 

When using solution mixing, the highest variation in terms of *T_g_* is an increase of 10 °C, obtained using 1 wt % MWCNT purified by treatment with strong acids. Comparing with non-purified filler at the same loading, the increase is 5 °C higher. This is explained by purified MWCNT having stronger interfacial interactions with PLA matrix, imposing increased restriction to the mobility of macromolecular chains, and therefore rising *T_g_*. Also, *T_d_* (decomposition temperature) presents an increase of 10 °C for purified materials [88]. For *T_m_*, the higher increase is of 16 °C for 0.3 and 1 wt % MWCNT-PCL (functionalized with poly(caprolactone)) incorporated in PLA aligned fibers by sonication in dichloromethane and electrospinning. Also, *T_c_* decreases more than 10 °C, due to MWCNT inducing heterogeneous crystallization [145]. However, the higher decrease in *T_c_* (<20 °C), is obtained by Moon et al. [138], with the incorporation of 3–10 wt % MWCNT, with a length of about 2000 µm. In literature, the degradation temperatures of the polymeric materials determined by TGA are presented in different terms. For example, as *T_di_* (beginning of thermal degradation), *T_d_*_5_ (decomposition temperature for 5 wt % loss), and *T_d_*_50_ (decomposition temperature for 50% weight loss). For *T_di_*, the highest increase is of 20 °C, obtained incorporating 2.5 wt % MWCNT-COOH (carboxylated with strong acids) by sonication in PLA dispersed in dichloromethane and THF, followed by vacuum drying and compression molding [146]. Considering *T_d_*_50_, the best result is an increase of 1–3 °C, in a work above described [145].

GBM incorporation also induces changes on thermal properties of PLA. For *T_g_*, an increase of 7 °C was obtained sonicating 0.4 wt % GNP in PLA films prepared by solvent casting [135]. The highest increases in *T_m_* have been of 5 °C, for samples obtained by compression molding of PLA with 0.5 wt % GO grafted with PLA, produced by vacuum drying a dispersion in chloroform [150]. Significant decrease in *T_c_*, of 20 °C, is observed for PLA with 2 wt % GO, obtained by solvent mixing [153]. Thermal stability of PLA has been shown to improve with addition of GBM. 2 wt % GONSs (graphene oxide nanosheets) increases *T_di_* by 16 °C in samples produced by solvent mixing [156]. Also, *T_d_*_5_ is increased by 11 °C sonication of 0.2 wt % GNSs (graphene nanosheets) in PLA dispersed in DMF, dried under vacuum to produce composites [152]. Finally, *T_d max_* (*T* of maximum degradation rate) increases 33 °C for PLA filled with TRG, produced by solution mixing [154]. Chemical modifications of MWCNT are reported to increase thermal properties of the composites. For example, directly comparing with PLA/MWCNT(non-modified), the incorporation of 1 wt % MWCNT grafted with PLA in the same PLA matrix, results in increases of about 3 °C in *T_g_* and decreases of 9 °C in *T_c_* [141]. Treatment with strong acids followed by silanization of SWCNT [144], which are incorporated in PLA at loading ranging from 0.1 and 3 wt %, results in increases of about 5 °C in *T_g_*. 

Concerning composites produced by melt-blending, the highest increases in *T_g_* are of 5–6 °C, for PLA micro-fibers with 3 wt % MWCNT to PLA [184]. Also, *T_c_* is observed to decrease at most 12 °C with incorporation of 0.5 and 2 wt % MWCNT [170]. Chieng et al. [167], study on the thermal properties of PLA/PEG (9:1) blends with addition of 0.1–1 wt % GNP, reveals no variations on *T_g_*, *T_m_*, and *T_c_*. However, *T_di_*, *T_max_*, and *T_50_*, increase by 56, 53, and 44 °C, respectively, for 0.5 wt % loadings. 

In situ polymerization of l-lactide in presence of TRG in amounts from 0.01 to 2 wt % result in considerable increases on *T_g_*, *T_m_*, and *T_dmax_*. For example, at 2 wt % loading, increases of 5, 14, and 18 °C are obtained, respectively [176]. In a different work reporting in situ polymerization of l-lactide, covalent functionalization of GO with both 1,4-butanediol, and polyhedral silsesquioxane results in increases in *T_g_* (18, 20 °C), *T_c_* (15, 8 °C), *T_m_* (7, 5 °C), and *T_d5_* (23, 11 °C) comparing with PLA/GO composites at 1 wt % loadings [177]. 

### 5.4. Biological Properties

**Figure 5:**
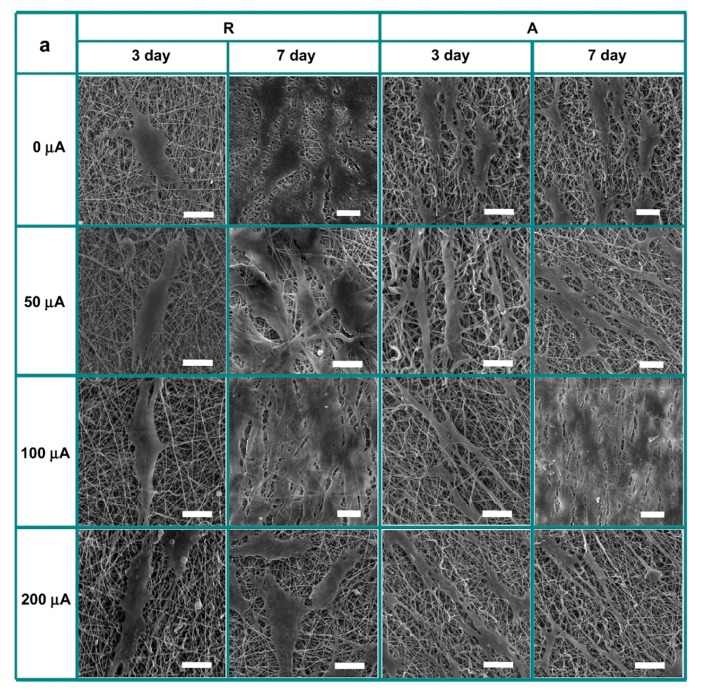

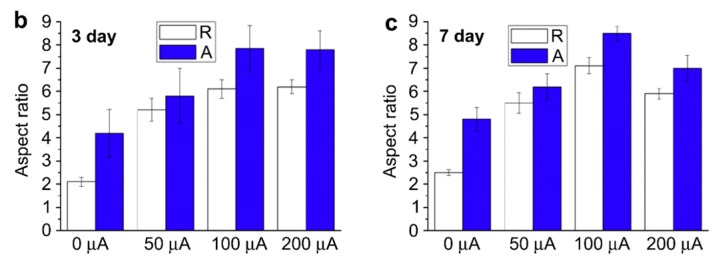
Scanning electron microscopy images of osteoblasts cultured on random (R) and aligned (A) nanofiber meshes of PLA/multi-walled carbon nanotubes (MWCNT)-ox 3 wt % produced by solution mixing followed by electrospinning, without or with electrical stimulation 0-200 μA (**a**); Osteoblast elongation is presented as the aspect ratio (**b**,**c**). Scale bars represent 30 μm [191].

Most nanomaterials may present toxicity at concentrations above a certain threshold when in isolated form, i.e., when not incorporated in a polymer matrix [40,186]. Biocompatibility of the composites must be tested when considering uses as biomaterials. Table 4 shows that PLA/CBN composites (films and nanofibers) do not tend to decrease in vitro metabolic activity of several cell types, or cause increases up to 40% until 72 h incubations. Also, the selection of production method used (melt blending or solvent mixing followed by casting, doctor blading, spin coating or electrospinning), does not seem to influence cell proliferation. For long term incubations, McCullen et al. [187] shows that scaffolds of PLA with 1 wt % MWNTs do not to influence metabolic activity of adipose-derived human mesenchymal stem cells (hMSCs) at 7 days. At 14 days, cells present increased metabolic activity and longitudinal alignment induced by the scaffolds. Sherrell et al. [188] reports PLGA (1:1) with a surface layer of graphene applied by CVD to increase PC-12 cells average length of neurites by 2.5 fold when electrical stimulated. Also, hemocompatibility improvements are reported with both incorporation of 0.4 wt % GNP by solvent mixing followed by doctor blading [149] and 4 wt % MWCNT by extrusion followed by injection molding [189] in PLA. In the last case, MWCNT alignment is associated with decreased platelet adhesion and activation. Thus, alignment seems to be generally benefit for biocompatibility. The bioeffectiveness of electrical stimulation together with nanofibers and its fillers alignment is confirmed by Shao et al. [183], which cultures osteoblasts at the surface of PLA/MWCNT-ox (3 wt %) produced by solution mixing followed by electrospinning. They observe improvements in cell elongation (190%) and metabolic activity (20%) for random nanofibers (d ≈ 250 nm) under DC 100 μA, comparing to unstimulated controls. For aligned fibers the previous values increase by 90 and 40%, respectively. The aspect ratio is higher for the latter, comparing with random stimulated fibers (Figure 5). Finally, An et al. [190] find that PLA composite films and nanofibers with 3 wt % PU and 5 wt % GO almost completely suppress *Escherichia coli* and *Staphylococcus aureus* growth after 24 h, not affecting MC3T3-E1 cells metabolic activity. This effect is attributed to GO potentially inducing oxidative stress or physical disruption on bacteria.

In an in vivo study, Kanczler et al. [192] observe that PLA-CB 0.1 wt % scaffolds seeded or not with fetal femur-derived cells, when implanted in a murine critical-size femur segmental defect model aid the regeneration of bone defect. Pinto et al. [193] report both PLA/GNP-M5 (2 wt %) and CNT-COOH (0.3 and 0.7 wt %) to be biocompatible, both in vitro and in vivo (2 weeks subcutaneous implantation in C57Bl/6 mice). Also, PLA/GNP-M5 and C 0.25 wt % composites have not release toxic products after 6 months degradation in phosphate-buffered saline at 37 °C [180]. This is relevant considering that long-term biocompatibility must be assured for safe PLA/CBN composites implantation.

## 6. Conclusions

Both CNT and GBM nanofillers are effective at improving PLA thermo-mechanical and electrical properties. However, lower amounts of GBM (0.1–1 wt %) are usually needed when comparing with CNT (0.25–5 wt %). Melt-blending is less reported than solution mixing for production of PLA/CBN composites, maybe because it implies use of specialized equipment. Moreover, results show that melt blending suffers from some drawbacks, since viscous shear is less effective than solvent sonication for promoting exfoliation/deagglomeration of CBN. In situ polymerization is the least reported technique, with further research being needed to demonstrate its advantages over the previous production methods.

Surface modifications of CBN can be used to improve compatibility with a polymer matrix. Functionalization with carboxyls is the most common and effective procedure to improve CNT dispersibility and compatibility with PLA. Some authors refer that purification with strong acids introduces polar groups in the carbon surface, which results in positive interaction with PLA. Besides straightforward chemical oxidation of CBN, other chemical modifications which lead to better performance after incorporation in PLA, comparing with non-modified CBN, include reaction with isocyanates, polyols, or silanes, and grafting with polymers (ethylene glycol, poly(caprolactone), poly(methyl methacrylate), poly(vinyl pyrrolidone), and PLA).

When comparing reduced and oxidized forms of GBM as PLA fillers, like rGO and GO, only in the case of increasing electrical conductivity the reduced forms show clearly better performance. 

Based on the available data, no relation can be determined between CBN morphological properties (size, length, and diameter) and the composites performances. 

The alignment of PLA/CNT fibers, has been shown to improve electrical conductivity. Electrical properties also improve with the increase of the amount of CBN incorporated.

Concerning biological properties, the composite production process does not influence cell metabolic activity, which does not decrease comparing to non-filled PLA. Furthermore, increases up to 40% in cell viability can be induced by GBM incorporation. Improvements in hemocompatibility are achieved with incorporation of both CNT and GBM. Also, both fiber/filler alignment and electrical stimulation, improve cell metabolic activity and elongation. Short term in vivo studies reveal PLA/CBN composites to be biocompatible, and no release of toxic degradation products is found up to 6 months in vitro degradation of PLA/GBM composites. Incorporation of GO has lead to suppression of *Escherichia coli* and *Staphylococcus aureus* growth, without compromising the composite biocompatibility. However, there is still no information on antimicrobial activity of these composites on other types of microorganisms or with other types of GBM. Also, long-term in vivo biocompatibility of PLA/CBN composites needs to be assured prior to their clinical use.

Some other relevant topics for future research include obtaining a better understanding of how the fillers physico-chemical properties, and their alignment inside the polymer matrix, affect the composites properties. In situ polymerization of PLA in presence of CBN is a not well developed topic, being worthwhile of further exploration due to the potential for optimization of the degree of interaction and dispersion of CBN in the polymer matrix. Mechanical milling is an increasingly interesting technique for mixing filler nanoparticles with a polymer matrix, but has not yet been reported for producing PLA/CBN composites. This is expected to change in the near future. Finally, emerging technologies, like 3D printing, will surely contribute to the conception of materials appropriate for the broad potential applications of PLA/CBN composites. 

## Figures and Tables

**Table 1:** Mechanical properties of PLA/CBN composites in comparison with non-modified PLA. Production methods and CBN characteristics.

**Method**	**Procedure**	**CNT Characteristics**	**CNT Content (wt %)**	**Mechanical Properties** **Relative to Neat Polymer** *ΔE*: maximum Young’s modulus improvement *ΔE’*: maximum storage modulus improvement *Δσ_max_*: maximum tensile strength improvement	**References**
**Solution mixing**	Sonication in chloroform and DMF, electrospinning	MWCNT Diameter (d) 15 ± 5 nm Length (l) 5–20 µm 95% purity Produced by plasma enhanced CVD	MWCNT: 0.25, 0.5, 1	*ΔE*↑372% (0.25 wt %)	[147]
	Sonication in chloroform, drying and compression molding (200 °C, 150 Kgf cm^−2^, 15 min)	MWCNT d not given l ± 2000 µm	MWCNT: 0.5, 3, 5, 10	*ΔE*↑150% (5 wt %)	[138]
	Sonication in chloroform, film casting	Unzipped CNT (uCNT) Diameter 30 nm l = 10 µm 95% purity	uCNT: 1, 2, 3, 4, 5	*ΔE’*↑14% (3 wt %)	[139]
	PLA was modified with benzoyl chloride and pyridine (PLAm), then acid chloride groups were added by reaction with thionyl chloride and triethylamine, then fMWCNT were added and the mixture centrifuged and filtered to remove excess filler and salts. Finally, sonication in chloroform and film casting was performed	MWCNT functionalized with COOH using Fenton reactant and then reacted with SOCl_2_ and ethylene glycol (fMWCNT) d = 9.5 nm l = 1.5 µm 95% purity	Not clear	*ΔE*↑17%, *Δσ_max_*↑8% (comparing to PLAm)	[140]
	Sonication in chloroform, coagulation with methanol, filtration, vacuum drying, and compression molding (180 °C)	MWCNT (thermal CVD, d = 10–15 nm, l = 10–20 µm, 95% purity) MWCNT carboxyl-functionalized (MWCNT-COOH) by H_2_SO_4_ 1:3 HNO_3_, 3 h, 120 °C MWCNT grafted with PLA (MWCNT-*g*-PLA): MWCNT-COOH + l-lactide, 12 h, 150 °C, + tin(II) chloride, 20 h, 180 °C, under vacuum, filtration, vacuum drying	MWCNT: 1 MWCNT-COOH: 1 MWCNT-*g*-PLA: 0.1, 0.2, 0.5, 1, 5	PLA/MWCNT-*g*-PLA: *ΔE*↑32%, *Δσ_max_*↑47% (1 wt %)	[141]
	Solution mixing in chloroform, drying and compression molding (180 °C)	MWCNT grafted with PLLA after reaction with SOCl_2_ and ethylene glycol (MWCNT--PLLA) Dimensions not given 95% purity	MWCNT and MWCNT-*g*-PLLA: 0.1, 0.2, 0.4, 0.6, 0.8, 1.2	PLA/MWCNT: *ΔE*↑46%, *Δσ_max_*↑9% (1.2 wt %) PLA/MWCNT-*g*-PLLA: *ΔE*↑86%, *Δσ_max_*↑13% (1.2 wt %)	[142]
	Solution mixing in chloroform, filtered, washed, dried under vacuum, and compression molded (180 °C, 500 psi)	MWCNT, MWCNT-COOH (both as in [101]), and MWCNT grafted with PLA chains of 122–530 g mol^−1^ by ring open polymerization (MWCNT-*g*-PLA530). d = 10–15 nm l = 10–20 µm 95% purity	MWCNT-COOH: 1 MWCNT-*g*-PLA530: 1	PLA/MWCNT-COOH: *ΔE*↑4%, *Δσ_max_* = 9% PLA/MWCNT-*g*-PLA530: *ΔE*↑44%, *Δσ_max_* = 44%	[143]
	Solution mixing in THF, vacuum drying, thermal compression	SWCNT (d < 2 nm, l = 5–15 µm, 95% purity) treated with 3:1 H_2_SO_4_/HNO_3_ (A-SWCNT), and functionalized (1:2 *v*/*v*) with 3-isocyanatoporpyl triethoxysilane (IPTES)—A-SWCNT-Si	SWCNT, A-SWCNT and A-SWCNT-Si: 0.1, 0.3, 0.5, 1, 3	PLA/SWCNT: *ΔE’*↑20% PLA/A-SWCNT: *ΔE’*↑33% PLA/A-SWCNT-Si: *ΔE’*↑67% (3 wt % for all conditions)	[144]
	Sonication in dichloromethane and THF, vacuum drying, and compression molding (190 °C)	MWCNT (d = 9–20 nm, l = 5 µm) functionalized with 3:1 H_2_SO_4_/HNO_3_ (MWCNT-COOH)	MWCNT-COOH: 0.5, 1, 2.5 MWCNT: 2.5	PLA/MWCNT-COOH: *ΔE*↑80%, *ΔE’*↑35%, *Δσ_max_*↑28% (2.5% wt %) PLA/MWCNT: *ΔE*↑25%, *ΔE’*↓6%, *Δσ_max_* (not reported) (2.5 wt %)	[146]
**Melt blending**	Internal mixer (180 °C, 50 rpm, 5 min) with and without transesterification with Ti(OBu)_4_, compression molding (180 °C)	MWCNT (l = 1–10 µm) functionalized with HNO_3_ (120 °C, 40 min)—MWCNT-COOH, and modified with DCC and stearyl alcohol (MWCNT-C_18_OH)	PC: MWCNT/PLA CNT-C_18_OH/PLA PC-18T: MWCNT-C_18_OH/PLA transesterified 0.5, 1.5, 3	(3 wt %) PLA/PC: *ΔE*↑73%, *ΔE’*↑34% PLA/PC-18: *ΔE*↑74%, *ΔE’*↑44% PLA/PC-18T: *ΔE*↑88%, *ΔE’*↑76%	[160]
	Twin-screw extrusion (150–190 °C, 100 rpm), injection molding (160–190 °C) High-crystalline PLA (HC-PLA) and low-crystalline PLA (LC-PLA) were tested	MWCNT (l = 5–20 µm, d = 40–60 nm) functionalized with maleic anhydride (MWCNT-*g*-MA) at 80 °C, 4 h, +benzoyl peroxide	LC-PLA/MWCNT, HC-PLA/MWCNT and MWCNT-*g*-MA: 0.25, 0.5, 0.75, 1, 2, 4	PLA/LC-PLA/MWCNT: *Δσ_max_*↑23% PLA/HC-PLA/MWCNT: *Δσ_max_*↑13% PLA/MWCNT-*g*-MA: *Δσ_max_*↑27% (4 wt % for all conditions)	[163]
	Twin-screw extrusion (180 °C, 150 rpm, 5 min), compression molding at 180 °C	MWCNT (d = 6–13 nm, l = 2.5–20 µm, specific surface area = 220 m^2^g^−1^) produced by CVD	MWCNT: 1.5, 3, 5	PLA/MWCNT: *ΔE’*↑28%, *Δσ_max_*↑27% (5 wt %)	[165]
	Twin-screw extrusion (160–190 °C)	Carboxyl–functionalized (MWCNT–COOH) d = 10–11 nm, l = 12–15 µm	MWCNT-COOH: 1	*ΔE* and *Δσ_max_*↑8% (1 wt %)	[181]
**Method**	**Procedure**	**GBM Characteristics**	**GBM Content (wt %)**	**Mechanical Properties** **Relative to Neat Polymer** *ΔE*: maximum Young’s modulus improvement *ΔE’*: maximum storage modulus improvement *Δσ_max_*: maximum tensile strength improvement	**References**
**Solution mixing**	Sonication in chloroform, casting and doctor blading GO was pre-dispersed in acetone while GNP was directly dispersed in chloroform	GNP grade M (commercial product) t = 6–8 nm, d ≈ 5 µm. GO (MHM) d ≈ 100 nm	GO and GNP: 0.2, 0.4, 0.6	PLA/GO: *ΔE*↑115%, *Δσ_max_*↑95% (0.3 wt %) PLA/GNP: *ΔE*↑156%, *Δσ_max_*↑129% (0.4 wt %)	[135]
	Sonication in chloroform, filtration, vacuum drying, compression molding (170 °C, 10 min)	GO (from natural graphite, MHM + lyophilization) d ≈ 300 nm GO-*g*-PLLA (GO + l-lactide (Sn(oct)_2_), filtration, vacuum drying)	GO and GO-*g*-PLLA: 0.5	PLA/GO: *Δσ_max_*↑51% PLA/GO-*g*-PLLA: *Δσ_max_*↑106%	[150]
	Stirring and sonication in DMF, coagulation with methanol, filtration, and vacuum drying	GO (MHM) from expandable graphite, chemically reduced with hydrazine, and lyophilized (GNSs—solvent free graphene nanosheets) t < 1 nm, d < 50 nm	GNSs: 0.2	*ΔE’*↑18%, *Δσ_max_*↑26%	[152]
	Sonication in DMF, coagulation with methanol, drying, compression molding (185 °C)	TRG (commercial product, t = few layer, d = hundreds of nm) TRG/PLA/Py-PLA: Py-PLA-OH (1-Pyrenemethanol + l-lactide, Sn(oct)_2_) + TRG (10:1)—sonication + PLA—coagulation and drying	TRG and TRG/PLA/Py-PLA: 0.25, 1	PLA/TRG: *ΔE’*↑1%–3%, *Δσ_max_*↑8% PLA/TRG/PLA/Py-PLA: *ΔE’*↑10%–15%, *Δσ_max_*↑19%	[154]
	Solution mixing in DMF, film casting	GO prepared according to MHM, reduced to rGO and functionalized with N-(aminoethyl)-aminopropyltrimethoxysilane (KH792)	rGO-KH792: 0.1, 0.2, 0.5	*ΔE’*↑1500% around the *T_g_* (0.5 wt %)	[157]
**Melt blending**	Twin-screw mixer (175 °C, 60 rpm, 8 min), compression molding at 180 °C	GO prepared by MHM and reduced with hydrazine and ammonia (rGO)t = 0.4–0.6 nm, d = 0.1–0.5 µm	rGO: 0.02, 0.04, 0.08, 0.2, 0.5, 1, 2	*ΔE’*↑27%, *Δσ_max_*↑40% (0.08 wt %) *ΔE’*↑54%, *Δσ_max_*↓40% (2 wt %)	[168]
	Internal mixer (160 °C, 25 rpm, 10 min), compression molding (160 °C, 10 min) *(Polymer was PLA/PEG 9:1 blend*)	GNP grade M15 (commercial product) t = 6–8 nm, d ≈ 15 µm	GNP-M15: 0.1, 0.3, 0.5, 0.7, 1	*ΔE’*↑84 and 70%, *Δσ_max_*↑20 and 33% (0.1 and 0.3 wt %) *(relative to pristine PLA/PEG blend)*	[167]
	Internal mixer (180 °C, 80 rpm, 10 min) Compression molding (180 °C)	GO (MHM) + SDS, ultrasounds, stirring 12 h, 25 °C Methylmethacrylate (MMA), stirring 12 h + ammonium persulfate (APS) 12 h, 80 °C + reduction with dimethyl hydrazine, 100 °C, 2 h (PFG—polymer-functionalized graphene nanoparticles) t = 2.4 nm	PFG: 1, 2, 3, 4, 5	*ΔE*↑80%, *Δσ_max_*↑10% (5 wt %)	[164]
	Internal mixer (180 °C, 50 rpm, 20 min) Compression molding (190 °C, 2 min, 150 Kg cm^−2^)	GNP grade M5 (t = 6–8 nm, d ≈ 5 µm) and C (t = up to 2 single layers, d < 2 µm) (commercial products)	GNP-M5 and C: 0.1, 0.25, 0.5	PLA/GNP-M5: *ΔE*↑14%, *Δσ*_max_↑6% (0.25 wt %) PLA/GNP-C: *ΔE*↑14%, *Δσ*_max_↑20% (0.25 wt %) The incorporation of both fillers prevented mechanical properties decay after 6 months degradation	[180,182]
**In situ polymerization**	Sonication of l-lactide + filler in toluene, addition of Tin(II)-2-ethylhexanoate under N_2_, stirring at 110 °C, 3 days	Expanded graphite (MHM) to GO GO-functionalized: GO + TDI + 1,4-butanediol, 80 °C, 24 h GO-*g*-POSS: GO + POSS—polyhedral oligomeric silsesquioxane + DMAP—4-(dimethylaminopyridine) + EDC—*N*-(3-dimethylamino-propyl-*N*’-ethylcarbodiimide), 2 days, room temperature, N_2_ (dimensions not given)	GO-functionalized, GO-*g*-POSS, GO+POSS (physical mixture): 1	PLA/GO-functionalized: *ΔE’*↑1%, *Hardness*↑14% PLA/GO-*g*-POSS: *ΔE’*↑33%, *Hardness*↑45% PLA/GO + POSS: *ΔE’*↑29%, Hardness↑36%	[177]

**Table 2:** Electrical properties of PLA/CBN composites in comparison with non-modified PLA. Production methods and CBN characteristics.

**Method**	**Procedure**	**CNT Characteristics**	**CNT Content (wt %)**	**Electrical Properties** σ: electrical conductivity *ρ_□_*: sheet resistance (PLA *σ* ≈ 1 × 10^−16^ S m^−1^, *ρ_□_* ≈ 5 × 10^12^ Ω sq^−1^) [106,122]	**References**
**Solution mixing**	Sonication in chloroform, drying and compression molding (200 °C, 150 Kgf cm^−2^, 15 min)	MWCNT Diameter (d) not given Length (l) = ±2000 µm	MWCNT: 0.5, 3, 5, 10	*σ* = 1.8 × 10^−3^ and 3.5 × 10^−3^ S m^−1^ (3 and 10 wt %)	[138]
	Sonication in chloroform, coagulation with methanol, filtration, vacuum drying, and compression molding (180 °C)	MWCNT (thermal CVD, d = 10–15 nm, l = 10–20 µm, 95% purity) MWCNT carboxyl-functionalized (MWCNT-COOH) by H_2_SO_4_ 1:3 HNO_3_, 3 h, 120 °C MWCNT grafted with PLA (MWCNT-*g*-PLA): MWCNT-COOH + l-lactide, 12 h, 150 °C, + tin(II) chloride, 20 h, 180 °C, under vacuum, filtration, vacuum drying	MWCNT: 1 MWCNT-COOH: 1 MWCNT-*g*-PLA:0.1, 0.2, 0.5, 1, 5	PLA/MWCNT: *ρ_□_* = 1 × 10^12^ Ω sq^−1^ (for 0.1 and 0.2 wt % is similar to PLA), 1 × 10^5^ and 1 × 10^4^ Ω sq^−1^ (0.5 wt %, and 1–5 wt %) PLA/MWCNT-*g*-PLA: *ρ_□_* = 1 × 10^12^ Ω sq^−1^ (0.1–5 wt %—always similar to PLA)	[141]
	Solution mixing in chloroform, drying and compression molding (180 °C)	MWCNT, MWCNT grafted with PLLA after reaction with SOCl_2_ and ethylene glycol (MWCNT-*g*-PLLA) Dimensions not given 95% purity	MWCNT and MWCNT-*g*-PLLA: 0.1, 0.2, 0.4, 0.6, 0.8, 1.2	PLA/MWCNT: *σ* = 2 × 10^−13^ S m^−1^ (0.1–0.4 wt %), 3 × 10^−9^ S m^−1^ (0.6 wt %), and 2 × 10^−5^ S m^−1^ (1.2 wt %) PLA/MWCNT-*g*-PLLA: *σ* = 2 × 10^−13^ S m^−1^ (0.1–0.4 wt %), 5 × 10^−13^ S m^−1^ (0.6 wt %), and 3 × 10^−8^ S m^−1^ (1.2 wt %) Increases with filler amount	[142]
	Solution mixing in chloroform, filtered, washed, dried under vacuum, and compression molded (180 °C, 500 psi)	MWCNT, MWCNT-COOH (both as in [101]), and MWCNT grafted with PLA chains of 122–530 g mol^−1^ by ring open polymerization (MWCNT-*g*-PLA122–530). d = 10–15 nm l = 10–20 µm 95% purity	MWCNT-COOH: 1 MWCNT-*g*-PLA122-530: 1	PLA/MWCNT-COOH: *ρ_□_* = 1 × 10^5^ Ω sq^−1^ PLA/MWCNT-*g*-PLA112-530: *ρ_□_* = 2 × 10^6^, 2 × 10^12^, and 1 × 10^12^ Ω/sq (122, 250, 530 g mol^−1^)	[143]
	Sonication in THF, vacuum drying, thermal compression	MWCNT (d = 8–15 nm, l = 50 µm) purified by sonication with H_2_SO_4_ and HNO_3_ at 50 °C, filtration, and washing	MWCNT purified/non-purified: 1, 3, 5, 7	PLA/MWCNT purified: *σ* = 4 × 10^−9^, 1 × 10^−9^, and 2 × 10^−6^ S m^−1^ (1, 5, and 7 wt %) PLA/MWCNT non-purified: *σ* = 7 × 10^−11^, 2 × 10^−8^, and 5 × 10^−8^ S m^−1^ (1, 5, and 7 wt %) Increases with filler amount	[88]
	Solution mixing in THF, vacuum drying, thermal compression	SWCNT (d < 2 nm, l = 5–15 µm, 95% purity) treated with 3:1 H_2_SO_4_/HNO_3_ (A-SWCNT), and functionalized (1:2 *v*/*v*) with 3-isocyanatoporpyl triethoxysilane (IPTES)—A-SWCNT-Si	SWCNT, A-SWCNT and A-SWCNT-Si: 0.1, 0.3, 0.5, 1, 3	PLA/SWCNT: *σ* = 2 × 10^−16^, 3 × 10^−9^, and 5 × 10^−8^ S m^−1^ (0.3, 1, 3 wt %) PLA/A-SWCNT-Si: *σ* = 5 × 10^−15^, 5 × 10^−8^, and 2 × 10^−6^ S m^−1^ (0.3, 1, 3 wt %) Increases with filler amount	[144]
	MWCNT-ox (HCl, 2 h at 25 °C + HNO_3_, 4h at 110 °C) Nanofibers (MWCNT-ox sonicated in DMF 2 h + SDS, adding to PLA in dicloromethane, 1 h sonication before electrospinning)	MWCNT (l = 10–20 µm, d = 10–20 nm) Nanofibers (PLA ≈ 400 nm, PLA/MWCNT-ox ≈ 250 nm)	PLA/MWCNT-ox (3 wt %) random (R) and aligned (A) nanofibers: 1, 2, 3, 4, 5 wt %	PLA/MWCNT-ox-R: *ρ_□_* = 1 × 10^4^, 5 × 10^2^ Ω sq^−1^ (3 and 5 wt %) PLA/MWCNT-ox-A: *ρ_□_* = 5 × 10^3^, 1 × 10^2^ Ω sq^−1^ (3 and 5 wt %) Increases with both fillers amount	[183]
**Melt blending**	Internal mixer (180 °C, 50 rpm, 5 min) with and without transesterification with Ti(OBu)_4_, compression molding (180 °C)	MWCNT (l = 1–10 µm) functionalized with HNO_3_ (120 °C, 40 min)—MWCNT-COOH, and modified with DCC and stearyl alcohol (MWCNT-C_18_OH)	PC: MWCNT/PLA PC-18: MWCNT-C18OH/PLA PC-18T: MWCNT-C18OH/PLA transesterified 0.5, 1.5, 3	PLA/PC: *ρ_□_* = 2 × 10^7^, 3 × 10^6^, and 3 × 10^5^ Ω sq^−1^ (0.5, 1.5, 3 wt %) PLA/PC-18: *ρ_□_* = 8 × 10^5^, 9 × 10^4^, and 1 × 10^−1^ Ω sq^−1^ (0.5, 1.5, 3 wt %) PLA/PC-18T: *ρ_□_* = 5 × 10^12^, 9 × 10^5^, and 9 × 10^−2^ Ω sq^−1^ (0.5, 1.5, 3 wt %)	[160]
	Twin-screw extruder (180, 215 and 250 °C; 100, 200 and 500 rpm; 5 min) 1st—masterbatch production 2nd—dilution of masterbatches and composites production	MWCNT d = 9.5 nm l = 1.5 µm 90% purity	MWCNT: 0.5, 0.75, 1, 2	*σ* is below 2.5 × 10^−1^ S m^−1^ (0.5–2 wt %) slightly decreasing with filler wt % increase	[162]
	Twin-screw extrusion (150–190 °C, 100 rpm), injection molding (160–190 °C) High-crystalline PLA (HC-PLA) and low-crystalline PLA (LC-PLA) were tested	MWCNT (l = 5–20 µm, d = 40–60 nm) functionalized with maleic anhydride (MWCNT-*g*-MA) at 80 °C, 4 h, + benzoyl peroxide	LC-PLA/MWCNT, HC-PLA/MWCNT and MWCNT-*g*-MA: 0.25, 0.5, 0.75, 1, 2, 4	LC-PLA/MWCNT: *ρ_□_* = 2 × 10^13^, 5 × 10^3^, and 5 × 10^2^ Ω sq^−1^ (0.5, 2, 4 wt %) HC-PLA/MWCNT: *ρ_□_* = 1 × 10^14^, 9 × 10^10^, and 8 × 10^10^ Ω sq^−1^ (0.5, 2, 4 wt %) LC-PLA/MWCNT-*g*-MA: *ρ_□_* = 3 × 10^2^, 2 × 10^2^, and 7 × 10^1^ Ω sq^−1^ (0.5, 2, 4 wt %)	[163]
	Twin-screw extrusion (180 °C, 150 rpm, 5 min), compression molding at 180 °C	MWCNT d = 6–13 nm, l = 2.5–20 µm, specific surface area = 220 m^2^ g^−1^ produced by CVD	MWCNT: 1.5, 3, 5	*σ* = 1 × 10^−9^, 1 × 10^−2^, and 1 S m^−1^ (1.5, 3, 5 wt %)	[165]
	Twin-screw extruder (180–220 °C, 500 rpm) Piston spinning (20, 50, 100 m min^−1^) to produce micro-fibers (220 °C, 3 min)	MWCNT d = 9.5 nm l = 1.5 µm 90% purity	MWCNT: 0.5, 1, 2, 3, 5	Extruded composites: *σ* = 4, 14, and 50 S m^−1^ (2, 3, 5 wt %) Fibers (3 wt %): *σ* = 50, 40, and 1 S m^−1^ (spinning speeds of 20, 50, and 100 m min^−1^)	[184]
**Method**	**Procedure**	**GBM Characteristics**	**GBM Content (wt %)**	**Electrical Properties** σ: electrical conductivity *ρ_□_*: sheet resistance (PLA *σ* ≈ 1 × 10^−16^ S m^−1^, *ρ_□_* ≈ 5 × 10^12^ Ω sq^−1^) [106,122]	**References**
**Solution mixing**	Sonication in DMF, coagulation with methanol, drying, compression molding (185 °C)	TRG (commercial product, t = few layer, d = hundreds of nm) TRG/PLA/Py-PLA: Py-PLA-OH (1-Pyrenemethanol + l-lactide, Sn(oct)_2_) + TRG (10:1)—sonication + PLA—coagulation and drying	TRG and TRG/Py-PLA-OH: 0.25, 1	PLA/TRG: *σ* = 1 × 10^−16^ and 1 × 10^−6^ S m^−1^ (0.25 and 1 wt %) PLA/TRG/PLA/Py-PLA-OH: *σ* = 1 × 10^−16^ and 1 × 10^−7^ S m^−1^ (0.25 and 1 wt %)	[154]
	Sonication in DMF, coagulation with methanol, drying, and compression molding (210 °C)	GO: from graphite flakes (modified Staudenmaier method) rGO-p: GO + Polyvinylpyrrolidone (1:5), sonication at 60 °C rGO-g: reduced by stirring with glucose in ammonia solution at 95 °C, 60 min Dimension not given	GO rGO-p rGO-g (0.5–2.5 vol %)	PLA/GO: *σ* = ↑6.5 × 10 ^−13^ S m^−1^ PLA/rGO-p: *σ* = ↑4.7 × 10 ^−8^ S m^−1^ PLA/rGO-g: *σ* = 2.2 S m^−1^ (for 1.25 vol % for all) Increases with filler amount	[155]
**Melt blending**	Twin-screw mixer (175 °C, 60 rpm, 8 min), compression molding at 180 °C	GO prepared according to MHM and chemically reduced to rGO. Thickness 0.4–0.6 nm and lateral dimension 0.1–0.5 mm.	rGO: 0.02, 0.04, 0.06, 0.2, 0.5, 1, 2	*σ* = 1 × 10^−13^ and 1 × 10^−9^ S m^−1^ (0.2 and 2 wt %) Increases with filler amount	[168]
	Internal mixer (180 °C, 80 rpm, 10 min)	GO (MHM) + SDS, ultrasounds, stirring 12 h, 25 °C Methylmethacrylate (MMA), stirring 12 h + ammonium persulfate (APS) 12 h, 80 °C + reduction with dimethyl hydrazine, 100 °C, 2 h (PFG—polymer-functionalized graphene nanoparticles) t = 2.4 nm	PFG: 1, 2, 3, 4, 5	*σ* = 5.6 × 10^−14^ and 2.6 × 10^−4^ S m^−1^ (1 and 5 wt %) Increases with filler amount	[164]
**In situ polymerization**	Ring-opening melt polymerization of lactide in presence of trGO	GO prepared according to MHM and thermally reduced to trGO Dimensions not given	TrGO: 0.01, 0.1, 0.5, 1, 1.5, 2	*σ* = 5 × 10^−6^ and 1.6 × 10^−2^ S m^−1.^ (1.5 and 2 wt %) Increases with filler amount	[176]

**Table 3:** Thermal properties of PLA/CBN composites in comparison with non-modified PLA. Production methods and CBN characteristics.

**Method**	**Procedure**	**CNTs Characteristics**	**CNTs Content (wt %)**	**Thermal Properties Relative to Neat Polymer**	**References**
**Solution mixing**	Sonication in chloroform, drying and compression molding (200 °C, 150 Kgf cm^-2^, 15 min)	MWCNT Diameter (d) not given Length (l) ≈ 2000 µm	MWCNT: 0.5, 3, 5, 10	*T_g_* (glass transition) ↓1–4 °C (3, 5 wt %) and = (10 wt %) *T_c_* (crystallization) ↓>20 °C (3, 5, 10 wt %) T_m_ (melting) = (3, 5, 10 wt %) *T_d_* (degradation) ↑10–20 °C (3, 5, 10 wt %)	[138]
	Sonication in chloroform, film casting	Unzipped CNT (uCNT) d = 30 nm l = 10 µm 95% purity	uCNT: 1, 2, 3, 4, 5	*T_g_* ↑7, 8 °C (3, 5 wt %) *T_m_* ↑5, 3 °C (3, 5 wt %)	[139]
	PLA was modified with benzoyl chloride and pyridine (PLAm), then acid chloride groups were added by reaction with thionyl chloride and triethylamine, then fMWCNT were added and the mixture centrifuged and filtered to remove excess filler and salts. Finally, sonication in chloroform and film casting was performed	MWCNT functionalized with COOH using Fenton reactant and then reacted with SOCl_2_ and ethylene glycol (fMWCNT). d = 9.5 nm l = 1.5 µm 95% purity	Not clear	*T_g_* (tanδ) ↑9 °C *T_di_* (beginning of thermal degradation) ↑80 °C	[140]
	Sonication in chloroform, coagulation with methanol, filtration, vacuum drying, and compression molding (180 °C)	MWCNT (thermal CVD, d = 10–15 nm, l = 10–20 µm, 95% purity) MWCNT carboxyl-functionalized (MWCNT-COOH) by H_2_SO_4_ 1:3 HNO_3_, 3 h, 120 °C MWCNT grafted with PLA (MWCNT-*g*-PLA): MWCNT-COOH + l-lactide, 12 h, 150 °C, + tin(II) chloride, 20 h, 180 °C, under vacuum, filtration, vacuum drying	MWCNT: 1 MWCNT-COOH: 1 MWCNT-*g*-PLA: 0.1, 0.2, 0.5, 1, 5	No significant changes in *T_m_* for all materials PLA/MWCNT: *T_g_* ↑3, *T_c_*↓3 °C (1 wt %) PLA/MWCNT-COOH: *T_g_* ↑2, *T_c_* ↓3 °C (1 wt %) PLA/MWCNT-*g*-PLA: *T_g_* ↑ 5–6 *T_c_* ↑1 ↓2, 6, 12, 19 °C (0.1, 0.2, 0.5, 1, 5 wt %)	[141]
	Sonication in dichloromethane, electrospinning	MWCNT (d = 8–15 nm, L—not given, 95% purity) were functionalized with -COOH by H_2_SO_4_ and HNO_3_ (3:1). Then, MWCNT-NH_2_ were produced reacting MWCNT-COOH with *N,N’*-dicyclohexylcarbodiimide (DCC). MWCNT-PCL were produced reacting 1 g MWCNT-NH_2_, 10 g PCL, and 20 g DCC	MWCNT-PCL(0.3, 0.5, 1, 3)/PLA aligned composite fibers	*T_d50_* (50% weight loss) ↑ 1–3 °C (0.3, 1 wt %) *T_g_* = (0.3, 1 wt %) *T_m_* ↑16 °C (0.3, 1 wt %) *T_c_* ↓13 °C and 12 °C (0.3, 1 wt %)	[145]
	Sonication in THF, vacuum drying, thermal compression	MWCNT (d = 8–15 nm, l = 50 µm) purified by sonication with H_2_SO_4_ and HNO_3_ at 50 °C, filtration, and washing	MWCNT purified/non-purified: 1, 3, 5, 7	PLA/MWCNT non-purified: *T_g_* ↑5–6 °C (1, 3, 5, 7 wt %) PLA/MWCNT purified: *T_g_* ↑10, 7, 5, 5 °C (1, 3, 5, 7 wt %) PLA/MWCNT non-purified vs. purified: *Td* ↑10, 11, 7, 8 °C (1, 3, 5, 7 wt %)	[88]
	Solution mixing in THF, vacuum drying, thermal compression	SWCNT (d < 2 nm, l = 5–15 µm, 95% purity) treated with 3:1 H_2_SO_4_/HNO_3_ (A-SWCNT), and functionalized (1:2 *v*/*v*) with 3-isocyanatoporpyl triethoxysilane (IPTES)—A-SWCNT-Si	SWCNT, A-SWCNT, and A-SWCNT-Si: 0.1, 0.3, 0.5, 1, 3	*T_d5_* (5 wt % loss) ↓ for PLA/SWCNT (poor interfacial interaction), = for PLA/A-SWCNT, and A-SWCNT-Si *T_g_*: (higher that pure PLA) PLA/SWCNT < PLA/A-SWCNT < PLA/A-SWCNT-Si (considering all loadings, increases are below 5 °C)	[144]
	Sonication in dichloromethane and THF, vacuum drying, and compression molding (190 °C)	MWCNT (d = 9–20 nm, l = 5 µm) functionalized with 3:1 H_2_SO_4_/HNO_3_ (MWCNT-COOH)	MWCNT-COOH: 0.5, 1, 2.5	*T_di_* ↑ 10–20 °C (0.5–2.5 wt %) *T_g_* ↑ 0, 1, 2 °C (0.5, 1, 2.5 wt %) *T_c_* ↑ 1, 2, 4 °C 0.5, 1, 2.5 wt %) *T_m_* ↑ 3, 4, 5 °C 0.5, 1, 2.5 wt %)	[146]
**Melt blending**	Internal mixer (180 °C, 50 rpm, 5 min) with and without transesterification with Ti(OBu)_4_, compression molding (180 °C)	MWCNT (l = 1–10 µm) functionalized with HNO_3_ (120 °C, 40 min)—MWCNT-COOH, and modified with DCC and stearyl alcohol (MWCNT-C_18_OH)	PC: MWCNT/PLA PC-18: MWCNT-C18OH/PLA PC-18T: MWCNT-C18OH/PLA transesterified 0.5, 1.5, 3	PLA/PC, PLA/PC-18—No change in *T_m_* PLA/PC-18T—2 melting peaks, 1 bellow *T_m_* for pristine PLA (low *M_w_* PLA from transesterification), other at the same *T_m_*	[160]
	Sonication in THF, vacuum drying + Microextruder (180 °C, 50 rpm, 5 min)	MWCNT (d = 9.5 nm, l = 1.5 µm) produced by catalytic carbon vapor deposition (CCVD)	MWCNT: 0.1, 1	*T_g_* ↑1 °C (0.1, 1 wt %)	[161]
	Twin-screw extruder (180, 215 and 250 °C; 100, 200 and 500 rpm; 5 min) 1st—masterbatch production 2nd—dilution of masterbatches and composites production	MWCNT d = 9.5 nm l = 1.5 µm 90% purity	MWCNT: 0.5, 0.75, 1, 2, 7.5, 15	Similar *T_g_* (7.5, 15 wt %)	[162]
	Twin-screw extruder (210 °C, 400 rpm), compression molding (210 °C)	MWCNT d = 5–20 nm l = 10 µm Specific surface area = 100–700 m^2^ g^-1^ CCVD	MWCNT: 0.5, 1, 2, 3, 5	*T_g_* ↓1, 2 °C (0.5, 1–5 wt %) *T_c_* ↓12, 10, 12, 7, 6 °C (0.5, 1, 2, 3, 5 wt %) *T_m_* ↓1, 2 °C (0.5–3, 5 wt %)	[170]
	Twin-screw extruder (180-220 °C, 500 rpm) Piston spinning to produce micro-fibers (220 °C, 3 min)	MWCNT d = 9.5 nm l = 1.5 µm 90% purity	MWCNT: 0.5, 1, 2, 3, 5	*T_g_*: pellet = (3 wt %) Fibers ↑ 5–6 °C (3 wt %)	[184]
**Method**	**Procedure**	**GBM Characteristics**	**GBM Content (wt %)**	**Thermal Properties Relative to Neat Polymer**	**References**
**Solution mixing**	Sonication in chloroform, casting and doctor blading GO was pre-dispersed in acetone while GNP was directly dispersed in chloroform	GNP grade M (commercial product) t = 6–8 nm, d ≈ 5 µm. GO (MHM) d ≈ 100 nm	GO and GNP: 0.2, 0.4, 0.6	PLA/GO: *T_g_* ↑3, 4, 3 °C (0.2, 0.4, 0.6 wt %) PLA/GNP: *T_g_* ↑6, 7, 5 °C (0.2, 0.4, 0.6 wt %) Similar *T_m_* for both GO and GNP	[135]
	Sonication in chloroform, filtration, vacuum drying, compression molding (170 °C, 10 min)	GO (from natural graphite, MHM + lyophilization) d ≈ 300 nm GO-*g*-PLLA (GO + l-lactide (Sn(oct)_2_), filtration, vacuum drying)	GO and GO-*g*-PLLA: 0.5	PLA/GO: *T_g_* ↑6 °C *T_m_* ↑3 °C PLA/GO-*g*-PLLA: *T_g_* ↑6 °C *T_m_* ↑5 °C	[150]
	Stirring and sonication in DMF, coagulation with methanol, filtration, and vacuum drying	GO (MHM) from expandable graphite, chemically reduced with hydrazine, and lyophilized (GNSs—solvent free graphene nanosheets) t < 1 nm, d < 50 nm	GNSs: 0.2	*T_d5_* ↑11 °C	[152]
	Sonication in DMF, film casting, vacuum drying	GO prepared according to Staudenmaier method (H_2_SO_4_ + HNO_3_ + KClO_3_) (dimensions not given)	GO: 0.5, 1, 2	(0.5, 1, 2 wt %) *T_c_* ↓9, 15, 20 °C *T_g_* similar	[153]
	Sonication in DMF, coagulation with methanol, drying, compression molding (185 °C)	TRG (commercial product, t = few layer, d = hundreds of nm) TRG/PLA/Py-PLA: Py-PLA-OH (1-Pyrenemethanol + l-lactide, Sn(oct)_2_) + TRG (10:1)—sonication + PLA—coagulation and drying	TRG and TRG/Py-PLA-OH: 0.25, 1	PLA/TRG: *T_d5_* ↓32 °C *T_d max_* (max. degradation) ↑33 °C PLA/TRG/PLA/Py-PLA: *T_d5_* ↓2 °C *T_d max_* ↑25 °C (loadings not clear)	[154]
	Sonication in DMF, coagulation with water, vacuum drying, compression molding (200 °C, 3 min)	Graphene oxide nanosheets—GONSs (MHM) from expandable graphite (t = few layer, d = 5–20 µm)	GONSs: 0.25, 0.5, 1, 2	(0.25, 0.5, 1, 2 wt %) *T_m1_* ↓1, 4, 0, 1 °C *T_m2_* ↓0, 1, 1, 1 °C *T_c_*↓3, 6, 2, 4 °C *T_di_* ↑2, 6, 11, 16 °C	[156]
	Sonication in DMF, film casting, vacuum drying	GNS (commercial product) t = 5–25 nm, d = 0.5–20 µm, specific surface area = 50 m^2^ g^−1^	GNS: 1	Similar *T_g_* and *T_m1_*_and *2*_ *T_c_* ↑3 °C	[157]
**Melt blending**	Internal mixer (160 °C, 25 rpm, 10 min), compression molding (160 °C, 10 min) *(Polymer was PLA/PEG 9:1 blend*)	GNP grade M15 (commercial product) t = 6–8 nm, d ≈ 15 µm	GNP-M15: 0.1, 0.3, 0.5, 0.7, 1	*(relative to pristine PLA/PEG blend)* (0.1, 0.3, 0.5, 1 wt %) *T_g_* ↓0, 0, 1, 1 *T_m_* ↑2, 4 ↓1, 1 *T_c_* ↑1, 2, 2, 1 *T_di_*, *T_d max_*, *T_50_* ↑56, 53, 44 °C (0.5 wt %)	[167]
**In situ polymerization**	Melt ring-opening polymerization of l-lactide in presence of TRG (Sn(oct)_2_, 170 °C, 4 h), filtration, vacuum drying	Natural graphite (MHM + lyophilization)—GO GO thermal reduction (1000 °C, 1 min) to TRG t = few layers	TRG: 0.01, 0.1, 0.5, 1, 1.5, 2	(0.01, 0.1, 0.5, 1, 1.5, 2 wt %) *T_g_* = ↑9, 6, 6, 7, 8, 5 °C *T_m_* = ↑11, 12, 13, 14, 14, 14 °C *T_d max_* = ↑4, 13, 10, 11, 16, 18 °C	[176]
	Sonication of l-lactide + filler in toluene, addition of Tin(II)-2-ethylhexanoate under N_2_, stirring at 110 °C, 3 days	Expanded graphite (MHM) to GOGO-functionalized: GO + TDI +1,4-butanediol, 80 °C, 24 h GO-*g*-POSS: GO + POSS—polyhedral oligomeric silsesquioxane + DMAP—4-(dimethylaminopyridine) + EDC—*N*-(3-dimethylamino-propyl-*N*’-ethylcarbodiimide), 2 days, room temperature, N_2_ (dimensions not given)	GO-functionalized, GO-*g*-POSS, GO+POSS (physical mixture): 1	PLA/GO-functionalized: *T_d5_* ↑8, *T_g_* ↓8, *T_c_* ↑14, *T_m_* ↓2 °C PLA/GO-*g*-POSS: *T_d5_* ↑31, *T_g_* ↑10, *T_c_* ↑29, *T_m_* ↑5 °C PLA/GO+POSS: *T_d5_* ↑19, *T_g_* ↑12, *T_c_* ↑22, *T_m_* ↑3 °C	[177]

**Table 4:** Biological properties of PLA/CBN composites in comparison with non-modified PLA. Production methods and CBN characteristics.

Method	Procedure	CBN Characteristics	CBN Content (wt %)	Biocompatibility Properties	References
**Solution mixing**	GO—MHM Nanofibers (l = 11–14 µm) electrospinning	GO (thickness (t) = 1.5 nm, length (l) ≈ 1 µm)	PLGA (1:1)/GO 1 and 2 wt % nanofibers	Cell metabolic activity (MA): (PLGA = 100%, PLGA/GO 1 wt % ≈ 102%, PLGA/GO 2 wt % ≈ 108%, 48 h) (PC 12 cells)	[191]
	GO—MHM Films (t ≈ 5 μm)—spin coating	GO (not found)	PLGA (1:1)/GO films	Cell MA: Small increase (≈ 10%) comparing to PLGA for PLGA/GO 2 wt % (48 h) (Hela cells)	[179]
	GO—MHM Nanofibers (diameter (d) = 0.3–1.3 µm) electrospinning	GO (few layer)	PLA/HA(10 wt %)/GO nanofibers	Cell MA: 1, 2 and 5 wt % GO ↑, comparing to PLA/HA (24 h) Only nanofibers with 5 wt % GO presented higher MA than PLA/HA (48 h) (MC3T3-E1 cells)	[185]
	GO—MHM Films (t = 25–65 µm) solvent mixing + doctor blading	GO (d ≈ 500 nm)	PLA/GO films (0.4 wt %)	Cell MA: No variations until 48 h, except for PLA/GO after 24 h (more 13% than pristine PLA) (Mouse embryo fibroblasts 3T3) Hemocompatibility: Less human platelets activated in PLA/GNP comparing with PLA in presence of plasma proteins	[149]
	GNP—commercial product Films (t = 25–65 µm) solvent mixing + doctor blading	GNP-M5 (t ≈ 6–8 nm, l ≈ 5 µm)	PLA/GNP films (0.4 wt %)		
	Graphene—CVD (chemical vapor deposition) Films (t = 25–65 µm) solvent casting over graphene	Graphene (t = 2 layers)	PLGA(1:1)/graphene surface layer	Cell MA: No significant changes until 4 days for PC-12 cells (rat adrenal gland pheochromocytoma) Cell differentiation: with electrical stimulation the average length of neurites increased 2.5-fold	[188]
	GO—MHM Films (dimensions not found)—solvent mixing + solvent casting Nanofibers (d ≈ 1 μm) electrospinning	GO (not found)	PLA/PU (3 wt %)/GO (5 wt %) films and nanofibers	Cell proliferation: not decreased (MC3T3-E1 cells) Antibacterial effect: *E. coli* and *S. aureus* growth 100% reduced at 24 h	[190]
	MWNTs—CVD Scaffolds (d = 0.7 μm, average porosity = 87%, void space = 89%)—electrospinning	MWNTs (l = 5–20 mm, d = 5–15 nm)	PLA/MWNTs (1 wt %) scaffolds	Cell MA: equal until day 7 and increased with MWNTs at day 14 (hMSCs) Cell morphology: MWNTs induced longitudinal alignment on cells at day 14	[187,189]
	MWCNT-ox (HCl, 2 h at 25°C + HNO_3_, 4 h at 110 °C) Nanofibers (MWCNT-ox sonicated in DMF 2h + SDS, adding to PLA in dicloromethane, 1h sonication before electrospinning) (PLA nanofibers, d ≈ 400 nm, PLA/MWCNT-ox nanofibers, d ≈ 250 nm)	MWCNT (l = 10–20 µm, d = 10–20 nm)	PLA/MWCNT-ox (3 wt %) random (R) and aligned (A) nanofibers	Cell MA: increased for osteoblasts at day 3 for PLA/MWCNT-ox (3 wt %) R—20% and A—40%, under DC = 100 μA Cell morphology: induced osteoblasts alignment at day 3 for PLA/MWCNT-ox (3 wt %) R—↑190% and A—↑90%, under DC = 100 μA	[183,187]
**Melt blending**	MWNTs—CVD Composites (dimensions not found)—extrusion + injection moldingAligned composites—mechanical stretching at 90 °C	MWNTs (l = 10–30 mm, d = 20–40 nm)	PLA/MWNTs (5, 10, 15 wt %) composites	Hemolysis: bellow standard permissible (5%) in all cases, decreases with MWNTs incorporation and alignment Kinetic clothing time: increases with MWNTs incorporation and alignment (best was PLA/MWNTs 5 wt % which increased time by 480%) Platelet adhesion and activation: decreases with MWNTs incorporation and alignment	[183,189]
	GNP (commercial product) Composites (t ≈ 0.5 mm) Melt blending + compression molding	GNP-C (t = up to 2 single layers, l < 2 µm) GNP-M5 (t ≈ 6–8 nm, l ≈ 5 µm)	PLA/GNP-C and M5 (0.25 wt %) composites	Comparing with PLA: similar cell adhesion and growth at the surface No release of toxic products after 6 months degradation in phosphate-buffered saline at 37 °C	[180]
	GNP (commercial product) CNT-COOH—CVD, shortened, surface oxidized Composites (t ≈ 0.5 mm) Melt blending + compression molding	GNP-M5 (t ≈ 6–8 nm, l ≈ 5 µm) CNT-COOH (l < 1 µm, d = 9.5 nm, <8% COOH content)	PLA/GNP-M5 (2 wt %) PLA/CNT-COOH (0.3 and 0.7 wt %)	Biocompatible, both *in vitro* (human fibroblasts, HFF-1) and in vivo (2 weeks subcutaneous implantation in C57Bl/6 mice)	[193]
**Laser sintering**	CB (carbon black)—not found Scaffolds (several shapes)—surface selective laser sintering	(CB) Carbon black (d = 360 nm, surface area = 100 m^2^ g^−1^)	SSLS-PLA/CB 0.1 wt % scaffolds	SSLS-PLA/CB 0.1 wt % scaffolds seeded or not with fetal femur-derived cells aided regeneration of murine bone defect	[192]
